# A Genetic Code Alteration Is a Phenotype Diversity Generator in the Human Pathogen *Candida albicans*

**DOI:** 10.1371/journal.pone.0000996

**Published:** 2007-10-03

**Authors:** Isabel Miranda, Rita Rocha, Maria C. Santos, Denisa D. Mateus, Gabriela R. Moura, Laura Carreto, Manuel A. S. Santos

**Affiliations:** Department of Biology, Centro de Estudos do Ambiente e do Mar (CESAM), University of Aveiro, Aveiro, Portugal; University of British Columbia, Canada

## Abstract

**Background:**

The discovery of genetic code alterations and expansions in both prokaryotes and eukaryotes abolished the hypothesis of a frozen and universal genetic code and exposed unanticipated flexibility in codon and amino acid assignments. It is now clear that codon identity alterations involve sense and non-sense codons and can occur in organisms with complex genomes and proteomes. However, the biological functions, the molecular mechanisms of evolution and the diversity of genetic code alterations remain largely unknown. In various species of the genus *Candida*, the leucine CUG codon is decoded as serine by a unique serine tRNA that contains a leucine 5′-CAG-3′anticodon (tRNA_CAG_^Ser^). We are using this codon identity redefinition as a model system to elucidate the evolution of genetic code alterations.

**Methodology/Principal Findings:**

We have reconstructed the early stages of the *Candida* genetic code alteration by engineering tRNAs that partially reverted the identity of serine CUG codons back to their standard leucine meaning. Such genetic code manipulation had profound cellular consequences as it exposed important morphological variation, altered gene expression, re-arranged the karyotype, increased cell-cell adhesion and secretion of hydrolytic enzymes.

**Conclusion/Significance:**

Our study provides the first experimental evidence for an important role of genetic code alterations as generators of phenotypic diversity of high selective potential and supports the hypothesis that they speed up evolution of new phenotypes.

## Introduction

A number of exceptions to the standard genetic code have been discovered in prokaryotic and eukaryotic organisms, involving nonsense-to-sense and sense-to-sense codon identity changes [1], [2]. Twenty five of such alterations have been recorded in mitochondrial genetic codes of metazoan, fungi, red algae, green plants, alveolates, stramenopiles, haptophtes and euglenozoans [3]. The most remarkable alterations involve metazoan arginine AGA and AGG (AGR) codons, which changed their identity to serine at the base of the phylogenetic tree, and later on to translation-stop in vertebrates and to glycine in urochordates [4], [5].

In bacteria and eukaryotic cytoplasmic systems, 18 genetic code alterations have also been recorded, but unlike in mitochondria, they involve, with one exception, nonsense-to-sense codon identity changes or codon unassignments (codons that vanished from genomes). For example, in *Micrococcus* spp., *Mycoplasma* spp. and *Pseudomicrothorax dubius* the AGA/AUA, CGG and UGA codons are unassigned, respectively. In *Bacillus subtilis* UGA codons are used to terminate mRNA translation (stop codons) and to insert tryptophan, creating readthrough proteins [6]. In various species of ciliates, 1 or 2 stop codons changed their identity to either glutamine (UAA and UAG), glutamate (UAA) or cysteine (UGA). Interestingly, these genetic code alterations apparently minimize nonsense errors arising from re-assembly of the ciliates fragmented genome [7]–[10].

Those genetic code alterations show that certain codons, namely codons that start with uridine (UNN) or adenosine (ANN), in particular stop (UAA, UAG or UGA), arginine (AGR), serine (AGY) and isoleucine (AUA) codons are more prone to identity changes than others. The only exception to this first codon base rule occurs in yeast mitochondria where cytosine starting codons (CNN), namely leucine CUN codons, changed their identity to threonine. Also, the leucine CUG codon altered its identity to serine in the cytoplasm of various *Candida* and *Debaryomyces* species [11]–[13]. These findings suggest that the strength of the interaction of the first codon-anticodon base pair plays an important role in the evolution of genetic code alterations. Indeed, the change of identity of UAA and UAG stop codons to glutamine in various ciliates involved first base misreading by glutamine tRNAs, which decode glutamine CAA and CAG codons [8], [14]. But, it also indicates that other forces beyond codon-anticodon interaction play important roles in codon identity redefinition.

The unexpected flexibility of the genetic code described above is further highlighted by insertion of selenocystein (21^st^ amino acid) in the active sites of prokaryotic and eukaryotic selenoproteins and insertion of pyrrolysin (22^sd^ amino acid) in the active site of monomethylamine methyltransferase of *Methanosarcina barkeri* [15], [16]. These expansions involving reprogramming of UGA and UAG stop codons, respectively, highlight the potential of genetic code alterations/expansions to generate functional innovation. This hypothesis is supported by recent artificial expansion of the genetic code through synthetic biology methodologies. Indeed, 49 non-natural amino acids have already been incorporated into *E. coli*, yeast and mammalian cells [17]–[20], to produce novel proteins of biotechnological and biomedical interest. This dramatic demonstration of genetic code flexibility also unveiled an extraordinary capacity of complex organisms to tolerate partial codon identity redefinition [21].

We are using *C. albicans* as a model system to elucidate the evolution of genetic code alterations. In this case, a unique serine tRNA (tRNA_CAG_^Ser^) decodes leucine CUG codons as serine [22]–[24]. However, the tRNA_CAG_^Ser^ is recognized by both leucyl- and seryl-tRNA synthetases (LeuRS and SerRS) and it is aminoacylated *in vitro* with both serine (97%) and leucine (3%) [25]. This unusual dual aminoacylation of the tRNA_CAG_^Ser^ has been preserved to the present day [26], [27], raising the intriguing possibility that it may play a role in *C. albicans* biology. It also provides strong support for the hypothesis that codon identity redefinition is driven through codon decoding ambiguity [28], [29]. In here, we have reconstructed the early stages of the *C. albicans* genetic code alteration to shed new light into those questions. This partial reversion of CUG identity from serine back to leucine triggered morphogenesis, phenotypic switching, and up-regulated expression of genes involved in cell adhesion and hyphal development and increased secretion of proteinases and phospholipases. Important karyotype alterations were also observed. The overall data suggest that *C. albicans* CUG ambiguity is an important phenotypic diversity generator and highlight important and yet overlooked functional roles for genetic code alterations.

## Results

### Reverting CUG identity from serine back to leucine

**Figure 1:**
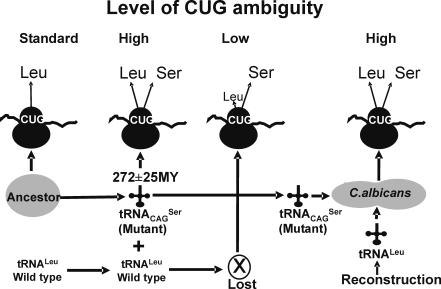
Reconstruction model for the *Candida* genetic code alteration. The ancestor of *Candida* decoded the CUG codon as leucine using a single leucine tRNA (tRNA^Leu^). This situation changed dramatically with appearance 272±25My of a mutant serine tRNA that acquired a 5′-CAG-3′anticodon (tRNA_CAG_^Ser^). The latter competed with the tRNA^Leu^ for decoding of CUG codons, inserting both leucine and serine, at high level, at CUG positions, on a proteome wide scale. Such ambiguity decreased over time due to disappearance of the tRNA^Leu^ gene, however charging of the tRNA_CAG_^Ser^ with leucine and serine prevented complete change of identity of the CUG codon from leucine to serine. In order to elucidate why CUG ambiguity was preserved in *C. albicans* and clarify whether CUG identity could be partially reverted from serine back to leucine, we have reconstructed the early stages of CUG identity change (high level of ambiguity) in *C. albicans* using *S. cerevisiae* tRNAs that decode CUG codons as leucine.

The CUG identity alteration from leucine to serine was initiated 272±25My ago through a mutant serine tRNA (tRNA_CAG_^Ser^), containing a 5′-CAG-3′ anticodon, which could decode CUG codons (see introduction) [26], [22]. Initially, this unique tRNA_CAG_^Ser^ competed with endogenous tRNA^Leu^, which decoded CUG codons as leucine [30], creating a new situation where both leucines and serines were inserted at CUG positions on a genome wide scale (Figure 1). For reasons not yet fully understood, the wild type tRNA^Leu^ disappeared from the *Candida* ancestor genome leaving CUG decoding exclusively to the mutant tRNA_CAG_^Ser^. Disappearance of the tRNA^Leu^ should have abolished the ambiguous status of CUG codons, however the tRNA_CAG_^Ser^ is recognized by both LeuRS and SerRS (see above), creating a serine tRNA that exists in 2 distinct forms, namely ser-tRNA_CAG_^Ser^ (charged with serine) and leu-tRNA_CAG_^Ser^ (charged with leucine). This ambiguous tRNA still exists in *C. albicans* [25].

**Figure 2:**
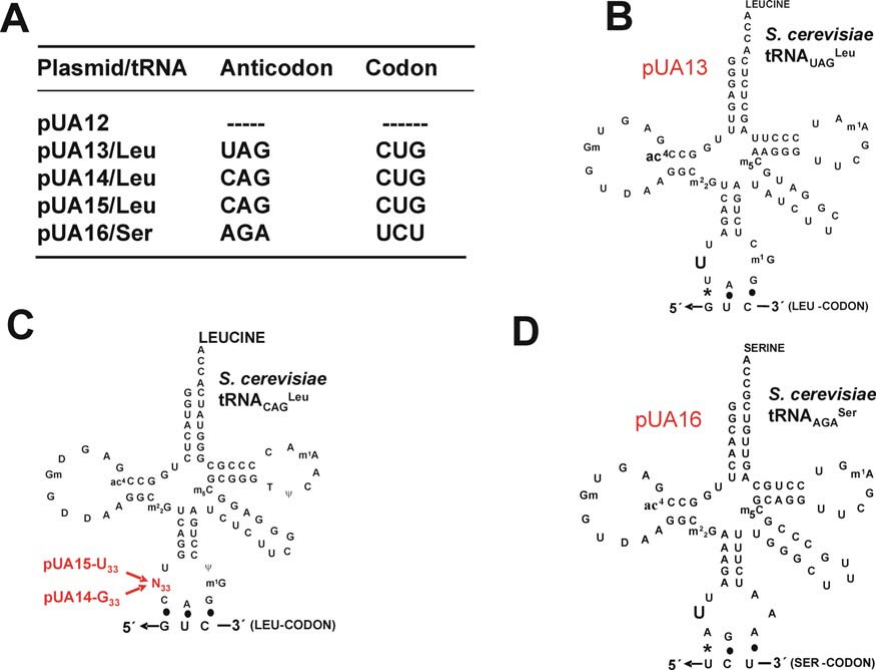
Transfer RNAs used in this study. In order to reconstruct the early stages of the CUG identity alteration in *C. albicans*, *S. cerevisiae* leucine tRNAs containing the anticodons UAG or CAG were expressed in C. *albicans*. A) The respective tRNA genes were cloned into plasmid pUA12, which is based on the *C. albicans* pRM1 vector. B) A leucine tRNA gene containing the near-cognate anticodon (5′-UAG-3′) for the CUG codon was used as a low decoding efficiency tRNA (pUA13). C) Two tRNA_CAG_^Leu^ genes, containing anticodons cognate for the CUG codon were used for higher CUG decoding efficiency, one contained G_33_ (pUA14; medium decoding efficiency) and the other contained U_33_ (pUA15; high decoding efficiency), in the anticodon-loop. D) The *S. cerevisiae* tRNA_AGA_^Ser^ gene was used as negative control (pUA16).

We have re-created in *C. albicans* (CAI-4 strain) the high ambiguity status of CUG codons that existed 272±25 My ago in the *Candida* ancestor (Figure 1). For this, we have expressed *Saccharomyces cerevisiae* wild type and mutant tRNAs, which decode CUG codons as leucine, in *C. albicans* (Figure 2A–D). These tRNAs^Leu^ competed with the novel tRNA_CAG_^Ser^ for CUG codons at the ribosome A-site, but were not lethal. We have hypothesized that such genetic manipulation would increase CUG ambiguity and could uncover phenotypes associated to the residual ambiguity (3%) [25] of CUG codons in *C. albicans*.

**Figure 3:**
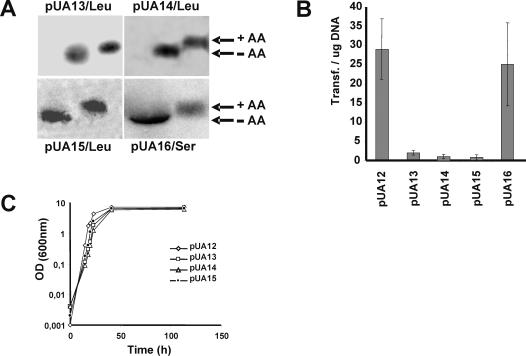
Expression of *S. cerevisiae* tRNA^leu^ in *C. albicans.* A) Aminoacylation *in vivo* in *C. albicans* of *S. cerevisiae* tRNA_UAG/CAG_^Leu^ and tRNA_AGA_^Ser^ was monitored by Acidic Page and Northern Blot analysis. For this, total tRNAs were extracted under acidic conditions from pUA13, pUA14, pUA15, and pUA16 clones and fractionated on an acidic polyacrylamide gel, as described in materials and methods. These gels separated deacylated (-AA) from aminoacylated tRNAs (+AA), which were detected using a tDNA_Leu/Ser_ probe labeled with [^32^P]. B) Transformation efficiencies of plasmids encoding tRNA^Leu^, which decoded the *C. albicans* serine CUG codons as leucine, was significantly lower that that of control plasmids (pUA12 and pUA16), indicating that the leucine tRNAs were slightly toxic. C) However, clones that survived the transformation procedure adapted readily to CUG ambiguity and showed growth rates similar to control clones (pUA12).

Three *S. cerevisiae* tRNA^Leu^ genes, namely a wild type tRNA_UAG_^Leu^ and two mutant tRNA_CAG_^Leu^ (containing U_33_ or G_33_ in the anticodon-loop), plus a control tRNA_AGA_^Ser^ (Figure 2B–D), were successfully expressed in *C. albicans* CAI-4 cells, as shown by Northern blot analysis of tRNAs fractionated by acidic-PAGE, which separates charged from uncharged tRNAs [31] (Figure 3A). This is in line with previous experiments that showed that the identity elements of *C. albicans* and *S. cerevisiae* LeuRS and SerRS are identical [32], [33]. Transformation efficiency of plasmids carrying the *S. cerevisiae* tRNA^Leu^ genes was lower than that of control plasmids (Figure 3B) containing no tRNA gene or containing a cognate serine decoder tRNA (pUA16) (Figure 2D). In other words, the tRNA^Leu^ were slightly toxic to *C. albicans*, which supported the hypothesis that they were fully functional and could incorporate leucine at CUG positions. Remarkably, clones that survived transformation showed no decrease in growth rate (Figure 3C), suggesting that they adapted to increased CUG ambiguity.

### Ambiguous CUG decoding generated important phenotypic diversity

**Figure 4:**
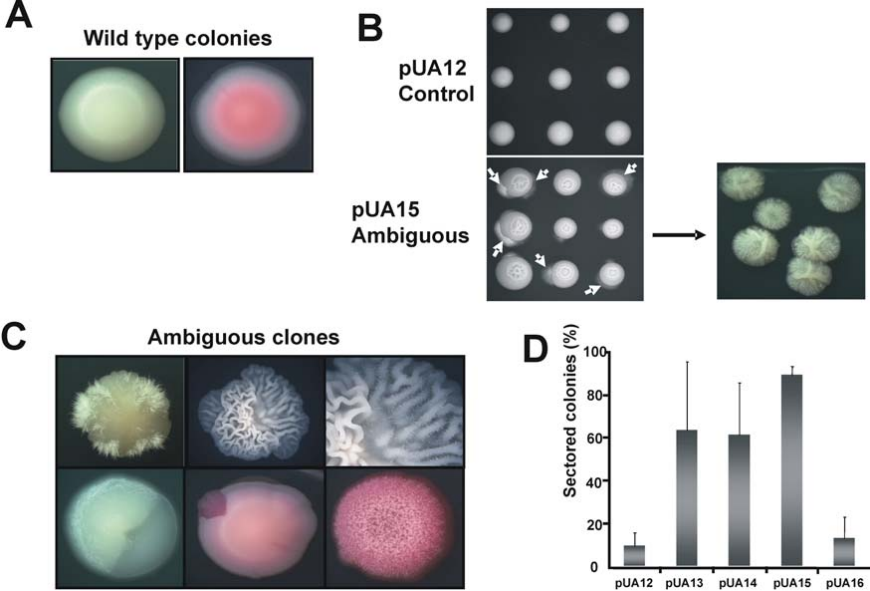
Ambiguous CUG decoding triggered morphogenesis and phenotypic switching. A) Smooth colony morphology of control clones growing on MM-uri-phloxin B (50µg/ml) agar plates. B) Ambiguous pUA15 clones formed long hyphae, even in absence of external inducers, just growing in MM-uri agar plates at 30°C. Similar results were obtained for pUA13 and pUA14 ambiguous clones (data not shown). D). Expression of *S. cerevisiae* tRNA^Leu^ in *C. albicans* also induced phenotypic switching, which is characterized by transition between different cell-phase forms, namely white-opaque and myceliated-unmyceliated, giving rise to sectored colonies. D) Phenotypic switching was quantified by counting sectored colonies grown in MM-uri, after 7 days of incubation at 30°C. For each plasmid, up to 10 clones were plated and 3000 colonies were screened.

Interestingly, expression of those *S. cerevisiae* tRNA^Leu^ genes in *C. albicans* triggered morphogenesis in both solid and liquid media (Figure 4). The pUA15 clones displayed extensive morphological variation (Figure 4B–C) and produced highly heterogenous cell populations containing elongated-ovoid cells, pseudohypha and true hypha (not shown). Some pUA15 clones produced hypha that occupied sectors or entire colonies. Notably, morphological events that gave rise to these phenotypes happened spontaneously without external inducing factors. As expected, control pUA12 and pUA16 clones had homogeneous morphology and formed smooth-white colonies similar to those of untransformed *C. albicans* CAI4 (Figure 4A). Similar results were obtained with clones pUA13 and pUA14 (data not shown). Apart from morphogenesis, CUG ambiguity also induced phenotypic switching, which is a *C. albicans* phenotype characterized by reversible induction of opaque or myceliated sectors in white smooth colonies [34]. High frequency of phenotypic switching (63–88%) was obtained for all clones expressing *S. cerevisiae* leucine tRNAs (pUA13-15), but not for control clones (pUA12 and pUA16) (Figure 4D).

**Figure 5:**
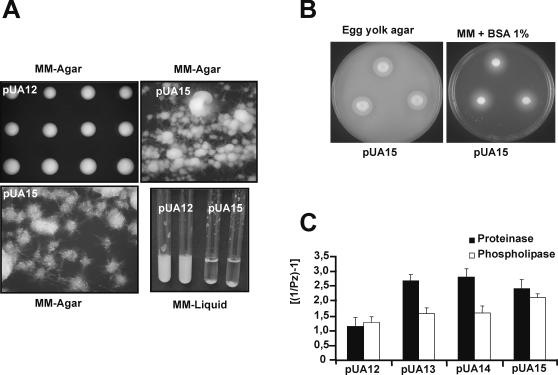
Increased CUG ambiguity resulted in higher hydrolytic activity and increased adhesion. A) Highly ambiguous cells (pUA15) exhibited strong adhesion phenotypes both in solid and liquid media. Adhesion to the solid agar surface resulted from cell-cell and cell-agar adhesion. In liquid media, cells showed a strong flocculation phenotype and sedimented even when grown with agitation (30°C for 2 days). B–C) Cells transformed with pUA13-14 (data not shown) and with pUA15 plasmids, had higher SAP and phospholipase activity than control cells, as determined by hydrolysis of BSA and egg yolk, respectively. Hydrolytic activity was quantified by measuring precipitation zones formed around the colonies, corrected by the colony diameter, in order to obtain Pz values.

CUG ambiguity also increased cell adhesion in liquid and solid media (Figure 5A), and once more, this phenotype was exacerbated in pUA15 clones, as they displayed strong flocculation in liquid media (Figure 5A). Interestingly, more than 50% of the genes involved in adhesion contain CUG codons. For example, the *ALS* gene family which encodes cell-surface glycoproteins that mediate adhesion to host surfaces [35], contain various CUG codons (3CUGs-*ALS2, ALS3, ALS8*; 4CUGs-*ALS4*; 5CUGs-*ALS1*; 11CUGs-*ALS6*; 12CUGs-*ALS9*; 18CUGs-*ALS7*). It is not yet clear whether the change of serine (polar) for leucine (hydrophobic) at CUG positions in the Als proteins is responsible for the flocculation and exacerbated agar adhesion observed in pUA15 clones. But, the strong adhesion phenotypes resulting from expression of *ALA1, EAP1* and members of the *C. albicans ALS* gene family, namely *ALS1* and *ALS5* in *S. cerevisiae*, support the hypothesis that the replacement of serines with leucines at CUG positions increases adhesion [36]–[41].

Finally, expression of *S. cerevisiae* tRNA^Leu^ (pUA15) in *C. albicans* increased production of extracellular hydrolases, namely secreted aspartic proteinases (SAP) and phospholipases, as determined on agar plates supplemented with BSA and egg yolk, respectively (Figure 5B,C). Hydrolysis of these substrates lead to formation of an halo of precipitated peptides around the colonies, indicative of SAP and phospholipase production [42]. Since adhesion, SAPs and phospholipases are important virulence attributes [43]–[45], those phenotypes are relevant to *C. albicans* pathogenesis and indicate, for the first time, that codon ambiguity and the *Candida* genetic code alteration may play a role in infection. It will be most interesting to put this hypothesis to experimental test as a positive result would clearly show that the negative impact of codon decoding ambiguity could be overcome by high selective potential of novel phenotypic diversity.

### Gene expression alterations

**Figure 6:**
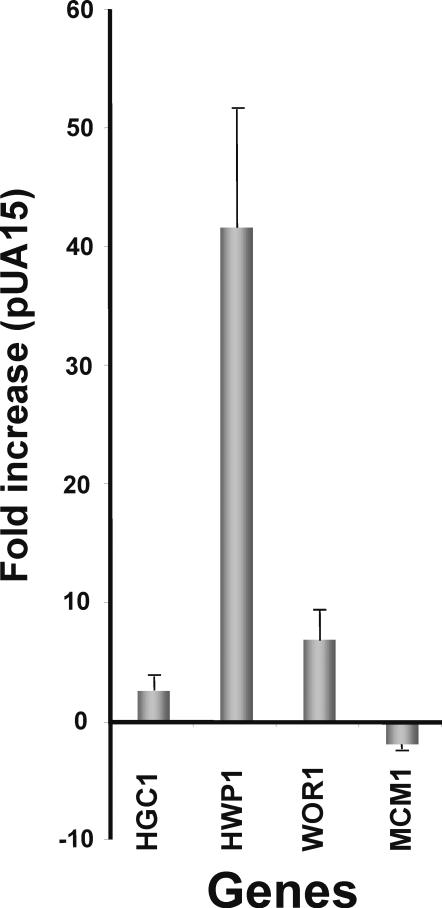
Increased CUG ambiguity up-regulated morphogenesis genes. Cells of pUA15 clones showed significant up-regulation of the *WOR1* (7.0±2.5) gene and hyphal-specific genes *CaHWP1* (41.76±9.96) and *HGC1* (2.64±1.12). Since *WOR1* increases the frequency of the white-opaque transition the very high percentage of opaque cells found in transformed clones may be a consequence of *WOR1* up-regulation. On the other hand, expression of the hypha-specific genes, *CaHWP1* and *CaHGC1,* supported the hypothesis that morphogenesis and hyphal growth triggered by CUG ambiguity resulted from expression of morphogenesis regulators. Induction of the *CaHWP1* gene was accompanied by repression of the *CaMCM1* (−1.84±0.44) gene, which controls cell morphology through the recruitment of other morphogenesis regulatory factors.

In order to elucidate how CUG ambiguity generated phenotypic diversity we have carried out gene expression profiling of *C. albicans* cells expressing the *S. cerevisiae* tRNA^Leu^ (pUA15 clones), using DNA microarrays. However, the diversity of phenotypes and permanent switching between phenotypes in culture (Figure 4) created cell variability and culture instability that prevented us from obtaining meaningful mRNA expression profiles (data not shown). To overcome this limitation, we have tried to stabilize some of the phenotypes on solid media, but, once again, this turned out to be very difficult to achieve due to very high switching between morphological forms. As the mRNA profiles represented average values of a variety of phenotypes, reproducibility was low and most genes failed to pass our microarray statistical filters. Despite this, we were able to detect 4 genes whose expression was consistently altered in the microarray data sets and were relevant to the pUA15 phenotypes, namely the hypha-specific G1 cyclin-related protein *HGC1* (2.64 fold), the hypha specific gene *HWP1* (41,76 fold), the white-opaque switch regulator *WOR1* (7 fold) and the transcription factor *MCM1* regulator of hyphal growth (-1.84 fold) (Figure 6). Expression of these genes was confirmed by Real Time PCR, as described in materials and methods.

The *WOR1* gene (7 fold up-regulated) is a master regulator of white-opaque switching and its expression induced the white-opaque phase transition [46], [47]. This provides a likely explanation for the strong induction of white-opaque switching in pUA15 clones, whose cells switch at high frequency. The *HWP1* gene (41,76±9,96 up-regulated) encodes a hyphal-specific cell wall mannoprotein, which is a substrate for mammalian transglutaminases and plays a crucial role in adhesion of *C. albicans* to epithelial cells [48]. Interestingly, *HWP1* expression was correlated with *MCM1* repression (-1.84 fold), confirming previous results that showed that expression of one of these genes represses expression of the other [49]. *MCM1* plays an important role in cell morphology and its expression is auto-regulated by a feedback control mechanism. Since both low and high Mcm1p levels lead to hyphal formation, it may act as a recruiting regulatory factor for morphogenesis in *C. albicans*. Depletion of Mcm1p induced transcription of *HWP1*, however no Mcm1p binding site was found in the *HWP1* promotor and it is not yet clear how the former activates transcription of the later. Finally, the *HGC1* gene was also up-regulated (2.64 fold). This gene is crucial for hyphal formation by promoting apical bud elongation and it is strongly induced during morphogenesis. It is not required for expression of hypha-specific genes (HSGs), like *HWP1,* but is positively regulated by the cAMP/PKA pathways and repressed by Tup1 and Nrg1 morphogenesis repressors [50].

### Increased CUG ambiguity increased *C. albicans* ploidy

**Figure 7:**
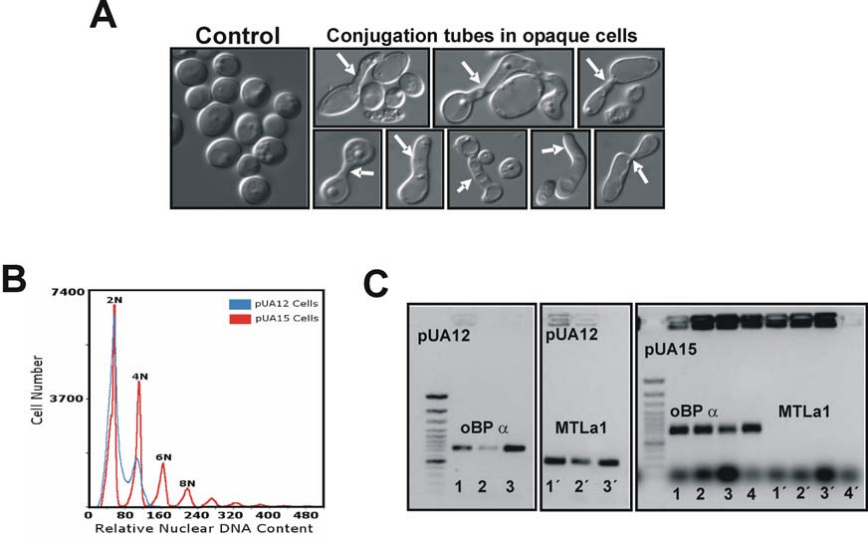
Ambiguous CUG decoding induced mating. A) In pUA15 transformed cells, the number of opaque cells was very high. This phenotype is most likely explained by up-regulation of the white-opaque master regulator *WOR1* gene (Figure 6). Since *C. albicans* white cells (most common form) are mating incompetent and opaque cells (rare cells) are mating competent, we have verified whether pUA15 opaque cells formed conjugation tubes and mating figures in liquid culture. Both were readily observed using optical microscopy (white arrows). B) In order to confirm that mating occurred, the DNA content of pUA15 cells was analyzed by flow cytometry. Since *C. albicans* is diploid, 4N cells were expected. Surprisingly, higher ploidies (6N, 8N) were also observed suggesting that the cultures had significant number of aneuploid and poliploid cells. C) White to opaque transition and mating induced by CUG ambiguity occurs due to MTL homozygosity. Since mating requires transition from the heterozygotic mating locus (MTL a/α) found in white cells, to the homozygotic configuration (MTLa/a or MTLα/α) found in opaque cells, detection of αα/αα cells supported the hypothesis that CUG ambiguity induced mating.

Up-regulation of the *WOR1* gene and the high percentage of opaque cells (mating competent) observed in pUA15 clones prompted the question of whether CUG ambiguity would induce mating in *C. albicans*. Indeed, pUA15 opaque cells formed conjugation tubes and mating figures which were readily observed by optical microscopy (Figure 7A). Furthermore, flow cytometry analysis of pUA15 clones identified sub-populations of 4N, 6N and 8N cells (Figure 7B), supporting the hypothesis that ambiguous *C. albicans* mated at high frequency. Since *C. albicans* is a diploid organism with a sexual life cycle [51], and considering that its mating locus (MTL) is heterozygotic in mating incompetent white cells (MTLa/α) and homozygotic in mating competent opaque cells (MTLa/a or MTLα/α) [52], the latter were isolated from pUA15 clones and were analysed for MTL homozygozity. All clones analysed were MTLα/α, suggesting that expression of tRNA^Leu^ (pUA15) induced biased MTLα/α homozygoty (Figure 7C), creating an excess of MTLα/α over MTLa/a cells. These results were in good agreement with up-regulation of the *WOR1* gene as its expression is controlled by the MTLa1-α2 heterodimer, which, in turn, controls white-opaque transition and mating competence [46], [47]. That is, switching from white-to-opaque phase required conversion of heterozygotic MTL to homozygotic MTL inducing mating competence [52]. Therefore, it is likely that MTL homozygoty induced by tRNA^Leu^ derepressed the *WOR1* gene, which, in turn, promoted the white-to-opaque transition and mating.

**Figure 8:**
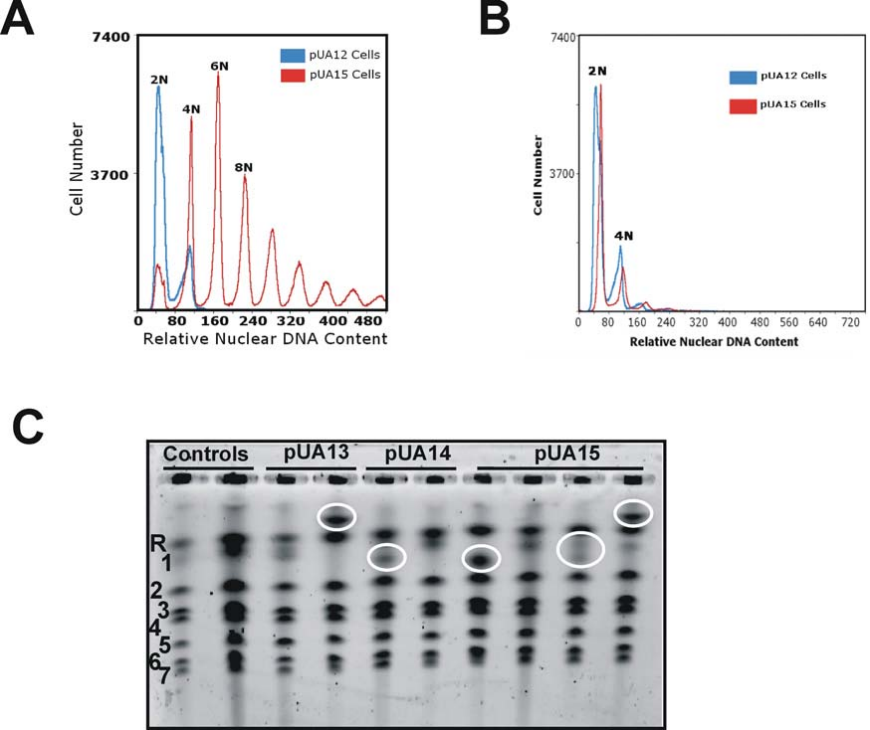
Ambiguous CUG decoding induced karyotype rearrangements and ploidy-shift. A) In ambiguous cell lines (pUA15) polyploidy was predominant and very high ploidy was often detected (>32N). Aneuploidy was also observed (6N). B) However, after plating cells several consecutive times on fresh agar chromosome numbers were reduced indicating that most cells returned to low ploidy (2N or 4N). Ploidy reduction after mating normally occurs by chromosome loss in *C. albicans* and it is likely that such mechanism also played a role in ploidy reduction in pUA15 transformed cells. C) CUG ambiguity also promoted extensive rearrangements of the R-chromosome (highlighted in white circles). Chromosomes were separated by PFGE on 0.6% agarose gels under the following conditions: 120–300 s for 24h at 80 V, then 420–900 s for 48 h at 80 V. The numbers 1–7 and R identify *C. albicans* chromosomes.

The presence of cells with high ploidy (6N and 8N) in pUA15 opaque cultures (Figure 7B), prompted us to monitor ploidy variability. Most clones showed increased ploidy ranging from 4N to 8N (data not shown), however very large cells with remarkably high ploidy (>64N) were also observed (Figure 8A). In general, ploidy variability between clones was higher than previously described [53], [54] and raised the question of whether those high chromosome numbers could return to 2N over time. Since wild type *C. albicans* undergoes a process of chromosome loss after mating, which decreases its ploidy from 4N back to 2N [55]–[57], we hypothesized that high ploidy in pUA15 clones could also be reduced. To test this hypothesis, clones with very high ploidy (large cells) were successively re-plated on fresh agar plates and their ploidy was monitored over time by flow cytometry. Indeed, ploidy reverted to 2N or 4N after several passages on fresh agar, confirming the above hypothesis (Figure 8B).

The above results also prompted us to check whether transformation of *C. albicans* with the pUA15 plasmid destabilized its karyotype. The *C. albicans* karyotype is characterized by frequent chromosome rearrangements, in particular of the chromosome R, which contains rDNA cistrons [58], [59]. We wondered whether the tRNA^Leu^ would affect rRNA metabolism and protein synthesis. Indeed, various rearrangements of the R-chromosome were readily observed (Figure 8C). In particular, the size of R-chromosomes increased in some clones, decreased in others and these rearrangements affected most cells (Figure 8C). It will now be most interesting to investigate whether CUG ambiguity affects ribosome assembly and the rate of protein synthesis.

## Discussion

The identity of CUG codons is variable in the genus *Candida*. Indeed, *C. glabrata* decodes CUGs as leucine, *C. cylindracea* changed their identity completely to serine and the other *Candida* species decode them ambiguously [13], [25], [60], [61]. These differences in CUG decoding arose from differences in the structure of the tRNA_CAG_^Ser^, which is the only cognate tRNA for CUG codons in *Candida*. Indeed, the various tRNA_CAG_^Ser^ have identity determinants for both SerRS and LeuRS [1]. For example, the *C. albicans* tRNA_CAG_^Ser^ contains identity elements for the LeuRS, namely m^1^G_37_ and the middle base of the anticodon (A_35_), but the discriminator base at position 73 (G_73_) is specific for SerRS and not for LeuRS (A_73_ discriminator) [62]. This base is critical for the recognition of tRNAs by aminoacyl-tRNA synthetases (aaRSs) and one would expect that the LeuRS would not recognize tRNAs with G_73_. Therefore, charging the tRNA_CAG_^Ser^ with leucine [25] suggests that the *Candida* LeuRS may have evolved a unique mode of recognition of its cognate tRNA^Leu^. Interestingly, the presence of a unique guanosine in the turn of the anticodon-loop (G-turn) of the tRNA_CAG_^Ser^ (G_33_), a conserved position occupied by U_33_ (U-turn), reduced leucylation efficiency of the tRNA_CAG_^Ser^ [25]. That is, recognition of the ancestral tRNA_CAG_^Ser^ by the LeuRS was efficient and G_33_ acted as a leucine identity anti-determinant. The reason for G_33_ selection is not yet clear, but one possibility is that it may have decreased the toxicity of the mutant tRNA_CAG_^Ser^ during the early stages of CUG identity alteration [27], [63].

The dual recognition of the tRNA_CAG_^Ser^ by the LeuRS and SerRS indicates that there are two forms of the tRNA_CAG_^Ser^ in the cytoplasm of *C. albicans*, namely Ser-tRNA_CAG_^Ser^ and Leu-tRNA_CAG_^Ser^, which are charged with serine and leucine, respectively. These 2 tRNAs generate ambiguity at CUG codons since they compete with each other for CUGs at the ribosome A-site. Interestingly, such CUG ambiguity was not constant over the 272±25My of evolution of the genetic code alteration (see introduction) [30]. It was high during the early stages of CUG identity change (when the tRNA_CAG_^Ser^ gene appeared), and decreased gradually due to elimination of the tRNA^Leu^ gene from the genome of the *Candida* ancestor [30]. Reconstruction of the high level of CUG ambiguity, which existed during the early stages of CUG identity alteration, provided the first insight on how the genetic chaos created at the onset of CUG identity change may have generated phenotypic diversity of evolutionary and adaptive relevance. In extant *C. albicans*, morphological variation alters cell surface antigens and it is likely that this pathogen uses its sophisticated capacity to generate morphological variation as a strategy to escape the immune system. Furthermore, secreted proteinases and phospolipases are important *C. albicans* virulence attributes and their increased activity in pUA15 clones may also be relevant to virulence. It will now be interesting to engineer stable high level mistranslation in *C. albicans* and test the virulence of the recombinant strains in mice models.

The high phenotypic diversity of pUA13, pUA14 and pUA15 clones and constant transition between phenotypes prevented us from carrying out a detailed study of the impact of CUG ambiguity on gene expression. However, the strong up-regulation of the hyphal specific gene (*HWP1*) and of the master regulator of the white-opaque transition (*WOR1*), may explain, at least in part, some of the phenotypes observed. As a hyphal specific gene (HSG), high *HWP1* expression, induced by CUG ambiguity, is in agreement with spontaneous morphogenesis events that generate filamentous cell populations. Furthermore, *HWP1* up-regulation may also increase adhesion since Hwp1p is a glycosylphosphatidylinositol cell wall adhesin (GPI-CWP) and mediates attachment of *C. albicans* cells to human endothelial and epithelial cells [44]. The observed adhesion phenotype (Figure 5A) may have also resulted from the combined up-regulation of *HWP1* and *WOR1* genes, since the later, previously known as *EAP2* (enhanced adhesion to polystyrene), mediates *C. albicans* and *S. cerevisiae* adhesion to polystyrene and epithelial cells [41].

Apart from its putative role in adhesion, up-regulation of *WOR1* may also explain the white-opaque phenotype since Wor1p is a transcriptional regulator of white-opaque switching [46], [47]. Indeed, Wor1p is present in very low amounts in white cells and accumulates in opaque cells. *WOR1* is repressed by the heterodimer MTL a1/α2 and is activated by Wor1p itself when cells become homozygous MTL aa/aa or MTL αα/αα. Increased accumulation of Wor1p triggered white-opaque switching and repressed its own transcription by a feedback regulatory mechanism [46], [47]. The up-regulation of the *HGC1* gene (Figure 6), which is a hypha-specific gene encoding a G1 cyclin-related protein, that plays a role in hyphal morphogenesis, was also significant. Since it is transcriptionally regulated by hypha-inducing rather than cell cycle signals [50], its up-regulation in ambiguous cells supports the hypothesis that it functions independently of other cell cycle cyclins.

CUG ambiguity also generated karyotype alterations and formation of polyploids and aneuploids. Ploidy-shift has been associated with chromosomal rearrangements [53], [54], [64] which also generates morphological variation [65]–[67], antifungal resistance [68], adaptation to alternative carbon sources [69], [70], or even with homozigosity of chromosome-V [71]. This suggests that part of the phenotypic diversity observed in pUA13, pUA14 and pUA15 clones may have resulted from karyotype alterations, or from a combination of up-regulation of the genes described above and karyotype destabilization. Since some clones did not have karyotype alterations (Figure 8C), but still displayed phenotypic variability, it is likely that the former does not play a main role in expression of phenotypic diversity. However, one should not exclude the hypothesis that genome destabilization contributes to exposure of hidden phenotypes through ambiguous CUG decoding. Finally, in *E. coli*, genetic code ambiguity induced by misreading tRNAs triggered translational stress-induced mutagenesis (TSM), due to synthesis of error-prone DNA polymerases [72]. This general mistranslation resulted in increased global error rates during DNA replication leading to heritable genetic changes. Since these hypermutagenic phenotypes result in rapid adaptation, an unexpected consequence of genetic code ambiguity (and genetic code alterations) is acceleration of genome variability and fast evolution of new phenotypes. This may explain evolution of the high plasticity of *Candida* morphology and the very high heterozigosity of its genome [73].

### Conclusions

Genetic code alterations pose important new biological questions whose answers remain elusive. It is now clear that a number of them evolved through codon decoding ambiguity, required significant structural change of protein synthesis machineries and reprogrammed codon usage [1], [5], [30]. However, codon decoding ambiguity is toxic, decreases fitness and may ultimately lead to cell death, as is the case in multicellular organisms [19], [74]. For these reasons, evolution of genetic code alterations through codon ambiguity is most puzzling. This study unveiled possible ways of overcoming the negative impact of codon ambiguity by high selective potential generated through generation of phenotypic diversity. The molecular mechanism used to generate such phenotypic diversity remains to be elucidated. However, the adaptive potential of the unveiled phenotypes strongly suggests that CUG ambiguity may have been preserved in *Candida* spp. as a novel generator phenotypic diversity. We have previously shown that codon ambiguity in *S. cerevisiae* creates a competitive edge under stress by inducing the general stress response and pre-adapting cells to sudden environmental changes [75]. Therefore, the toxicity of codon ambiguity is not an impediment to codon identity redefinition, supporting the hypothesis that codon misreading plays a critical role in the evolution of genetic code alterations and genetic code expansion.

## Materials and Methods

### Strains and growth conditions

Escherichia coli strain JM109 (recA1 SupE44 endA1 hsdR17 gyrA96 relA1 thi Δ(Lac-proAB) F'[traD36 proAB-lacI lacZ ΔM15] was used has a host for all DNA manipulations. Candida albicans CAI4 (ura3Δ::imm434/ura3::imm434) was grown at 30°C in YEPD (2% glucose; 1% yeast extract, 1% peptone). After transformation with pUA12, pUA13, pUA14, pUA15, and pUA16 plasmids, cells were grown in minimal medium lacking uridine (MM-uri) (0.67% yeast nitrogen base without amino acids, 2% glucose, 2% agar and 100µg/ml of the required amino acids). Solid MM-uri was supplemented with phloxin B (50µg/ml) in order to detect macroscopic growth of opaque cells.

### Plasmids

A multi-cloning site was inserted (*NruI/EcoRV*) in the low-copy *C. albicans* vector pRM1 [76]. The resulting vector was named pUA12. For heterologous expression of the *S. cerevisiae* tRNAs genes in CAI4, a genomic DNA fragment containing *S. cerevisiae* tRNA gene (700 bp), previously amplified by PCR, was cloned into the multi cloning site of pUA12 plasmid. *S. cerevisiae* Leu-tRNA_UAG_ and a 300 bp flanking region, upstream and downstream of the gene, was amplified with the following set of primers: 5′-CCGCTCGAGCGGCGACTGTCCAGACTTAGTAAAG CT-3′ and 5′-GCTCTAGAGCCCGCTGTCGCCAGCGTTAGC-3′. Genomic DNA containing *S. cerevisiae* tRNA_GAG_^Leu^ gene was used as a template for PCR amplification using the forward primers: 5′-GCTATGGGCCCGCCTCCGGGTAGTTGCAACGGTACTCTGG CCGAGTGGTCTAAGGCG-3′ and 5′-GCTATGGGCCCTAGTTGCAACGG TATCTGGCCGAGTGGTCTAAGGCGTCAGGTTCAGGTCC-3′; and reverse primer 5′-ATGCATAAAAACAAAATTTGTTGAAA-3′. These primers introduced a mutation in the first position of the anticodon changing it from 5′-GAG-3′ to 5′-CAG-3′ and allowed insertion of G or T at position 33 of the tRNA anticodon-loop. Upstream of this gene, a 250 bp fragment with the same sequence of the 5′ flanking *C. albicans* Ser-tRNA_CAG_ gene, was also inserted. This fragment was amplified by PCR from a *XhoI/ApaI* genomic DNA fragment, with the following primers: 5′-CCGCTCGAGCGGGTA TGCAATCGTTGTCTGTAATGTA-3′ and 5′-GCTATGGGCCCAAGCACAAA TGGTTATGACAATTG ATG-3′. The pUA16 control plasmid was constructed by inserting a *XhoI/AvaIII* genomic DNA fragment (600 bp), containing *S. cerevisiae* Ser-tRNA_AGA_ gene amplified by PCR with the following pair of primers 5′-CCGC TCGAGCGGGAGGATTCCTATATCCTTGAGGAG-3′ and 5′-GGCTCGATGCATG CCAGGAAGAAATACACTGC-3′, into the multicloning site. All DNA amplifications were carried out using a Mastercycle gradient (Eppendorf) and standard PCR protocols.

### Plasmid transformation

Transformation of *E. coli* was carried out as described by [77] CAI4 transformation was performed by the spheroplast method as described in the Manual for Preparation and Transformation of *Pichia pastoris* Spheroplasts (version A, Invitrogen).

### Northern Blot analysis

Acidic Northern Blot analysis was performed as described by Santos *et al.* (1996). For total tRNA extractions, 250 ml cultures grown overnight in YEPD or MM-uri medium were harvested at an OD_600_ of 0.7–0.9 and the pellets were frozen at −70°C overnight. Cells were resuspended in 5 ml lysis buffer (0.3M sodium acetate, pH 4.5, 10 mM EDTA), 1 vol. phenol equilibrated with sodium citrate pH 4.5 and baked glass beads. Cell suspension was vigorously shaken >30 seconds and incubated on ice for periods longer than 30 seconds, this procedure was repeated 8 times [78]. The aqueous phase containing RNAs was separated from the phenolic phase by centrifugation at 3200×*g* for 20 min at 4°C and then transferred to a new Falcon tube and re-extracted with fresh phenol. Aqueous phases containing RNAs were harvested by centrifugation at 3200×*g* for 20 min at 4°C and applied to a 20 ml DEAE-cellulose column equilibrated with 0.1 M sodium acetate pH 4.5. tRNAs were eluted with 0.1 M sodium acetate/1 M sodium chloride and precipitated with 2.5 vol. ethanol, resuspended in 10 mM sodium acetate pH 5.0/1 mM EDTA, and stored a −20°C. The deacylated tRNAs were obtained by incubation at 37°C for 1 h in 1 M Tris pH 8, 1 mM EDTA buffer [26].

tRNAs were fractionated at 4°C in 7.5% acrylamidde-8 M urea (30 cm long, 0.8 mm thick), buffered with 0.1 M sodium acetate pH 5.0. The 7.5% acidic gels were run at 300 V until bromophenol blue dye reached the bottom [31]. Fractionated tRNAs were transferred to nitrocellulose membranes (Hybond N, Amersham). Membranes were pre-hybridized for 6 h in a Hybridization oven at 50°C in 50% formamide, 5×SSC, 1% SDS, 0.04% Ficoll, 0.04% polyvinylpyrrolidone and 250 μg/ml sheared salmon sperm DNA [79]. Hybridization was performed overnight in the above buffer using probes labelled with [α-^32^P]dCTP by PCR, using standard protocols [80], except that the amount of dCTP was reduced from 100 to 50 mM and 5 nmol (30 μCi) 6000 Ci/mmol [α-^32^P]dCTP was added to the reaction mixture. In order to decrease the background level of free radioactivity, 50 PCR cycles were performed to decrease the amount of non-incorporated [α-^32^P]dATP. Membranes were washed at low stringency in 1×SSC, 0.5% SDS at 50°C or at high stringency in 0.1×SSC, 0.5% SDS at 65°C for 1 h. The membranes were exposed overnight with intensifying screens and developed using a Molecular Imager FX (Biorad).

### Switching frequencies and phenotypic characterization

*C. albicans* grown overnight at 30°C in MM-uri were serially diluted to 1000 cells per ml. Approximately 50 cells were plated onto fresh agar plates and then allowed to grow at 30°C for 7 days in a humidified incubator to prevent drying of the agar surface. Sectored colonies displaying atypical colonies were scored and the data was analyzed for statistical significance using ANOVA. Colonies were photographed using a Stemi 2000-C dissecting microscope equipped with AxioCam HRc camera and Axio Vision Software from Zeiss. Cells were photographed using a Zeiss MC80 Axioplan 2 light microscope.

### Real Time RT-PCR

Total RNA was prepared from *C. albicans* using hot acid phenol [81]. First-strand cDNA synthesis was carried out using the Superscript II RT kit from Invitrogen and its quantification was carried out in an Applied Biosystems 7500 Real Time PCR system using the SYBR Green I dye quantification assay (Power SYBR Green PCR master Mix). Primer concentrations were tested (0,2 to 0,4 µM) to ensure the lowest threshold cycle (C_T_) and the highest signal magnitude against the target template and to ensure non-specific product formation, resulting from primer dimmerization. After amplification, reactions were checked for presence of non-specific products through dissociation curve analysis. Each gene was quantified using 9 replicas of both control (pUA12) and ambiguous (pUA15) clones and a mean value was calculated. Outliers were rejected using critical values of Dixon's “Q” parameter at 95% confidence level [82].

### Determination of extra cellular hydrolytic activity

*C. albicans* strains were screened for the production of extra cellular phospholipase and secreted aspartic proteinase activity by growing cells on MM-uri agar supplemented with 10% egg yolk (Merck) and 10% bovine albumin (Sigma), respectively. A 3×2 µl suspension of 10^7^cells/ml in PBS was plated on the surface of the agar medium and left to dry at room temperature. The culture was then incubated at 30°C for 3 days, after which the diameter of the colony and the precipitation zone around the colony was determined. Three different clones of pUA12, pUA13, pUA14 and pUA15 transformed cells were tested twice. The experiment was carried out on two different occasions. The extra cellular hydrolase activity was calculated using the formula [(1/Pz)-1], where Pz value represents the hydrolyse zone, i. e., the cloudy-zone-around-plus-colony diameter divided by the colony diameter [83]. Data obtained was submitted to an ANOVA statistical test.

### Genome analysis

DNA content of *C. albicans* cells was determined using FACS analysis [84]. Karyotype analysis was performed using Pulsed-Field Gel Electrophoresis (PFGE) [85].

### MTL analysis

PCR analysis of the MTL configuration was carried out in pUA12 and pUA15 cells, through amplification of *oBPα* and *MTLa1* genes, respectively, using the following pairs of primers: 5′ GTGGTCAATGGAGCTGATAC 3′and 5′ ACATGTGGTCGCCCAACTCC 3′; 5′ TTGAAGCGTGAGAGGCAGGAG 3′ and 5′ ATCAATTCCCTTTCTCTTCGATTAGG 3′.

## References

[1] Santos MA, Moura G, Massey SE, Tuite MF (2004). Driving change: the evolution of alternative genetic codes.. Trends Genet.

[2] Miranda I, Silva R, Santos MA (2006). Evolution of the genetic code in yeasts.. Yeast.

[3] Knight RD, Landweber LF, Yarus M (2001). How mitochondria redefine the code.. J Mol Evol.

[4] Castresana J, Feldmaier-Fuchs G, Paabo S (1998). Codon reassignment and amino acid composition in hemichordate mitochondria.. Proc Natl Acad Sci U S A.

[5] Knight RD, Freeland SJ, Landweber LF (2001). Rewiring the keyboard: evolvability of the genetic code.. Nat Rev Genet.

[6] Lovett PS, Ambulos NP, Mulbry W, Noguchi N, Rogers EJ (1991). UGA can be decoded as tryptophan at low efficiency in Bacillus subtilis.. J Bacteriol.

[7] Caron F, Meyer E (1985). Does Paramecium primaurelia use a different genetic code in its macronucleus?. Nature.

[8] Harper DS, Jahn CL (1989). Differential use of termination codons in ciliated protozoa.. Proc Natl Acad Sci U S A.

[9] Sanchez-Silva R, Villalobo E, Morin L, Torres A (2003). A new noncanonical nuclear genetic code: translation of UAA into glutamate.. Curr Biol.

[10] Le Mouel A, Butler A, Caron F, Meyer E (2003). Developmentally regulated chromosome fragmentation linked to imprecise elimination of repeated sequences in paramecia.. Eukaryot Cell.

[11] Tuite MF, Santos MA (1996). Codon reassignment in Candida species: an evolutionary conundrum.. Biochimie.

[12] Jukes TH, Osawa S (1996). CUG codons in *Candida* spp.. J Mol Evol.

[13] Sugita T, Nakase T (1999). Non-universal usage of the leucine CUG codon and the molecular phylogeny of the genus Candida.. Syst Appl Microbiol.

[14] Hanyu N, Kuchino Y, Nishimura S, Beier H (1986). Dramatic events in ciliate evolution: alteration of UAA and UAG termination codons to glutamine codons due to anticodon mutations in two Tetrahymena tRNAs.. EMBO J.

[15] Hao B, Gong W, Ferguson TK, James CM, Krzycki JA (2002). A new UAG-encoded residue in the structure of a methanogen methyltransferase.. Science.

[16] Lee BJ, Worland PJ, Davis JN, Stadtman TC, Hatfield DL (1989). Identification of a selenocysteyl-tRNA(Ser) in mammalian cells that recognizes the nonsense codon, UGA.. J Biol Chem.

[17] Bacher JM, Ellington AD (2001). Selection and characterization of Escherichia coli variants capable of growth on an otherwise toxic tryptophan analogue.. J Bacteriol.

[18] Bacher JM, Crecy-Lagard V, Schimmel PR (2005). Inhibited cell growth and protein functional changes from an editing-defective tRNA synthetase.. Proc Natl Acad Sci U S A.

[19] Nangle LA, Motta CM, Schimmel P (2006). Global effects of mistranslation from an editing defect in mammalian cells.. Chem Biol.

[20] Chin JW, Cropp TA, Anderson JC, Mukherji M, Zhang Z (2003). An expanded eukaryotic genetic code.. Science.

[21] Hendrickson TL, Crecy-Lagard V, Schimmel P (2004). Incorporation of nonnatural amino acids into proteins.. Annu Rev Biochem.

[22] Santos MA, Perreau VM, Tuite MF (1996). Transfer RNA structural change is a key element in the reassignment of the CUG codon in Candida albicans.. Embo J.

[23] Santos MA, Tuite MF (1995). The CUG codon is decoded in vivo as serine and not leucine in Candida albicans.. Nucleic Acids Res.

[24] Ohama T, Suzuki T, Mori M, Osawa S, Ueda T (1993). Non-universal decoding of the leucine codon CUG in several Candida species.. Nucleic Acids Res.

[25] Suzuki T, Ueda T, Watanabe K (1997). The ‘polysemous’ codon--a codon with multiple amino acid assignment caused by dual specificity of tRNA identity.. Embo J.

[26] Santos MA, Keith G, Tuite MF (1993). Non-standard translational events in Candida albicans mediated by an unusual seryl-tRNA with a 5′-CAG-3′ (leucine) anticodon.. Embo J.

[27] Perreau VM, Keith G, Holmes WM, Przykorska A, Santos MA (1999). The Candida albicans CUG-decoding ser-tRNA has an atypical anticodon stem-loop structure.. J Mol Biol.

[28] Schultz DW, Yarus M (1996). On malleability in the genetic code.. J Mol Evol.

[29] Schultz DW, Yarus M (1994). Transfer RNA mutation and the malleability of the genetic code.. J Mol Biol.

[30] Massey SE, Moura G, Beltrao P, Almeida R, Garey JR (2003). Comparative evolutionary genomics unveils the molecular mechanism of reassignment of the CTG codon in Candida spp.. Genome Res.

[31] Varshney U, Lee CP, RajBhandary UL (1991). Direct analysis of aminoacylation levels of tRNAs in vivo. Application to studying recognition of Escherichia coli initiator tRNA mutants by glutaminyl-tRNA synthetase.. J Biol Chem.

[32] O'Sullivan JM, Davenport JB, Tuite MF (2001). Codon reassignment and the evolving genetic code: problems and pitfalls in post-genome analysis.. Trends Genet.

[33] O'Sullivan JM, Mihr MJ, Santos MA, Tuite MF (2001). Seryl-tRNA synthetase is not responsible for the evolution of CUG codon reassignment in Candida albicans.. Yeast.

[34] Soll DR (2002). Phenotypic Switching.. Candida and Candidiasis.

[35] Hoyer LL (2001). The ALS gene family of Candida albicans.. Trends Microbiol.

[36] Fu Y, Rieg G, Fonzi WA, Belanger PH, Edwards JE (1998). Expression of the Candida albicans gene ALS1 in Saccharomyces cerevisiae induces adherence to endothelial and epithelial cells.. Infect Immun.

[37] Rauceo JM, Gaur NK, Lee KG, Edwards JE, Klotz SA (2004). Global cell surface conformational shift mediated by a Candida albicans adhesin.. Infect Immun.

[38] Gaur NK, Smith RL, Klotz SA (2002). Candida albicans and Saccharomyces cerevisiae expressing ALA1/ALS5 adhere to accessible threonine, serine, or alanine patches.. Cell Commun Adhes.

[39] Gaur NK, Klotz SA, Henderson RL (1999). Overexpression of the Candida albicans ALA1 gene in Saccharomyces cerevisiae results in aggregation following attachment of yeast cells to extracellular matrix proteins, adherence properties similar to those of Candida albicans.. Infect Immun.

[40] Gaur NK, Klotz SA (1997). Expression, cloning, and characterization of a Candida albicans gene, ALA1, that confers adherence properties upon Saccharomyces cerevisiae for extracellular matrix proteins.. Infect Immun.

[41] Li F, Palecek SP (2003). EAP1, a Candida albicans gene involved in binding human epithelial cells.. Eukaryot Cell.

[42] Ibrahim AS, Mirbod F, Filler SG, Banno Y, Cole GT (1995). Evidence implicating phospholipase as a virulence factor of Candida albicans.. Infect Immun.

[43] Naglik JR, Challacombe SJ, Hube B (2003). Candida albicans Secreted Aspartyl Proteinases in Virulence and Pathogenesis.. Microbiol Mol Biol Rev.

[44] Sundstrom P (2002). Adhesion in Candida spp.. Cell Microbiol.

[45] Ghannoum MA (2000). Potential role of phospholipases in virulence and fungal pathogenesis.. Clin Microbiol Rev.

[46] Zordan RE, Galgoczy DJ, Johnson AD (2006). Epigenetic properties of white-opaque switching in Candida albicans are based on a self-sustaining transcriptional feedback loop.. Proc Natl Acad Sci U S A.

[47] Huang G, Wang H, Chou S, Nie X, Chen J (2006). Bistable expression of WOR1, a master regulator of white-opaque switching in Candida albicans.. Proc Natl Acad Sci U S A.

[48] Staab JF, Bradway SD, Fidel PL, Sundstrom P (1999). Adhesive and mammalian transglutaminase substrate properties of Candida albicans Hwp1.. Science.

[49] Rottmann M, Dieter S, Brunner H, Rupp S (2003). A screen in Saccharomyces cerevisiae identified CaMCM1, an essential gene in Candida albicans crucial for morphogenesis.. Mol Microbiol.

[50] Zheng X, Wang Y, Wang Y (2004). Hgc1, a novel hypha-specific G1 cyclin-related protein regulates Candida albicans hyphal morphogenesis.. EMBO J.

[51] Hull CM, Johnson AD (1999). Identification of a mating type-like locus in the asexual pathogenic yeast Candida albicans.. Science.

[52] Miller MG, Johnson AD (2002). White-opaque switching in Candida albicans is controlled by mating-type locus homeodomain proteins and allows efficient mating.. Cell.

[53] Iwaguchi SI, Kanbe T, Tohne T, Magee PT, Suzuki T (2000). High-frequency occurrence of chromosome translocation in a mutant strain of Candida albicans by a suppressor mutation of ploidy shift.. Yeast.

[54] Suzuki T, Hitomi A, Magee PT, Sakaguchi S (1994). Correlation between polyploidy and auxotrophic segregation in the imperfect yeast Candida albicans.. J Bacteriol.

[55] Bennett RJ, Johnson AD (2003). Completion of a parasexual cycle in Candida albicans by induced chromosome loss in tetraploid strains.. EMBO J.

[56] Magee BB, Magee PT (2000). Induction of mating in Candida albicans by construction of MTLa and MTLalpha strains.. Science.

[57] Hull CM, Raisner RM, Johnson AD (2000). Evidence for mating of the “asexual” yeast Candida albicans in a mammalian host.. Science.

[58] Lasker BA, Carle GF, Kobayashi GS, Medoff G (1989). Comparison of the separation of Candida albicans chromosome-sized DNA by pulsed-field gel electrophoresis techniques.. Nucleic Acids Res.

[59] Magee BB, Magee PT (1987). Electrophoretic karyotypes and chromosome numbers in Candida species.. J Gen Microbiol.

[60] Santos MA, Ueda T, Watanabe K, Tuite MF (1997). The non-standard genetic code of Candida spp.: an evolving genetic code or a novel mechanism for adaptation?. Mol Microbiol.

[61] O'Sullivan JM, Santos MA, Tuite MF (2002). Standard and nonstandard mRNA decoding in Candida.. Candida and Candidiasis.

[62] Breitschopf K, Gross HJ (1994). The exchange of the discriminator base A73 for G is alone sufficient to convert human tRNA(Leu) into a serine-acceptor in vitro.. EMBO J.

[63] Santos MA, Perreau VM, Tuite MF (1996). Transfer RNA structural change is a key element in the reassignment of the CUG codon in Candida albicans.. EMBO J.

[64] Selmecki A, Bergmann S, Berman J (2005). Comparative genome hybridization reveals widespread aneuploidy in Candida albicans laboratory strains.. Mol Microbiol.

[65] Rustchenko-Bulgac EP, Howard DH (1993). Multiple chromosomal and phenotypic changes in spontaneous mutants of Candida albicans.. J Gen Microbiol.

[66] Rustchenko-Bulgac EP, Sherman F, Hicks JB (1990). Chromosomal rearrangements associated with morphological mutants provide a means for genetic variation of Candida albicans.. J Bacteriol.

[67] Suzuki T, Kobayashi I, Kanbe T, Tanaka K (1989). High frequency variation of colony morphology and chromosome reorganization in the pathogenic yeast Candida albicans.. J Gen Microbiol.

[68] Perepnikhatka V, Fischer FJ, Niimi M, Baker RA, Cannon RD (1999). Specific chromosome alterations in fluconazole-resistant mutants of Candida albicans.. J Bacteriol.

[69] Rustchenko EP, Howard DH, Sherman F (1997). Variation in assimilating functions occurs in spontaneous Candida albicans mutants having chromosomal alterations.. Microbiology.

[70] Janbon G, Sherman F, Rustchenko E (1998). Monosomy of a specific chromosome determines L-sorbose utilization: a novel regulatory mechanism in Candida albicans.. Proc Natl Acad Sci U S A.

[71] Legrand M, Lephart P, Forche A, Mueller FM, Walsh T (2004). Homozygosity at the MTL locus in clinical strains of Candida albicans: karyotypic rearrangements and tetraploid formation.. Mol Microbiol.

[72] Dorazi R, Lingutla JJ, Humayun MZ (2002). Expression of mutant alanine tRNAs increases spontaneous mutagenesis in Escherichia coli.. Mol Microbiol.

[73] Jones T, Federspiel NA, Chibana H, Dungan J, Kalman S (2004). The diploid genome sequence of Candida albicans.. Proc Natl Acad Sci U S A.

[74] Lee JW, Beebe K, Nangle LA, Jang J, Longo-Guess CM (2006). Editing-defective tRNA synthetase causes protein misfolding and neurodegeneration.. Nature.

[75] Santos MA, Cheesman C, Costa V, Moradas-Ferreira P, Tuite MF (1999). Selective advantages created by codon ambiguity allowed for the evolution of an alternative genetic code in Candida spp.. Mol Microbiol.

[76] Pla J, Gil C, Monteoliva L, Navarro-Garcia F, Sanchez M (1996). Understanding Candida albicans at the molecular level.. Yeast.

[77] Sambrook J, Fritsch EF, Maniatis T (1989). Molecular cloning: a laboratory manual..

[78] Weygand-Durasevic I, Nalaskowska M, Soll D (1994). Coexpression of eukaryotic tRNASer and yeast seryl-tRNA synthetase leads to functional amber suppression in Escherichia coli.. J Bacteriol.

[79] Heitzler J, Marechal-Drouard L, Dirheimer G, Keith G (1992). Use of a dot blot hybridization method for identification of pure tRNA species on different membranes.. Biochim Biophys Acta.

[80] Innis MA, Gelfand DH, Sninsky JJ, White TJ (1990). PCR protocols: A Guide to Methods and Applications..

[81] Kohrer K, Domdey H (1991). Preparation of high molecular weight RNA.. Methods Enzymol.

[82] Rorabacher DB (1991). Statistical Treatment for Rejection of Deviant Values - Critical-Values of Dixon Q Parameter and Related Subrange Ratios at the 95-Percent Confidence Level.. Analytical Chemistry.

[83] Samaranayake LP, Raeside JM, MacFarlane TW (1984). Factors affecting the phospholipase activity of Candida species in vitro.. Sabouraudia.

[84] Fortuna M, Sousa MJ, Côrte-Real M, Leão C, Salvador A (2000). Cell Cycle Analysis of Yeasts.. Current Protocols in Cytometry.

[85] Chu WS, Magee BB, Magee PT (1993). Construction of an SfiI macrorestriction map of the Candida albicans genome.. J Bacteriol.

